# Phytocannabinoids: Useful Drugs for the Treatment of Obesity? Special Focus on Cannabidiol

**DOI:** 10.3389/fendo.2020.00114

**Published:** 2020-03-04

**Authors:** Patrycja Bielawiec, Ewa Harasim-Symbor, Adrian Chabowski

**Affiliations:** Department of Physiology, Medical University of Bialystok, Bialystok, Poland

**Keywords:** cannabidiol, diabetes, drugs, glucose metabolism, obesity, phytocannabinoids

## Abstract

Currently, an increasing number of diseases related to insulin resistance and obesity is an alarming problem worldwide. It is well-known that the above states can lead to the development of type 2 diabetes, hypertension, and cardiovascular diseases. An excessive amount of triacylglycerols (TAGs) in a diet also evokes adipocyte hyperplasia and subsequent accumulation of lipids in peripheral organs (liver, cardiac muscle). Therefore, new therapeutic methods are constantly sought for the prevention, treatment and alleviation of symptoms of the above mentioned diseases. Currently, much attention is paid to *Cannabis* derivatives—phytocannabinoids, which interact with the endocannabinoid system (ECS) constituents. Δ^9^-tetrahydrocannabinol (Δ^9^-THC) and cannabidiol (CBD) are the most abundant compounds of *Cannabis* plants and their therapeutic application has been suggested. CBD is considered as a potential therapeutic agent due to its anti-inflammatory, anti-oxidant, anti-tumor, neuroprotective, and potential anti-obesity properties. Therefore, in this review, we especially highlight pharmacological properties of CBD as well as its impact on obesity in different tissues.

## Introduction

A well-known ancient plant *Cannabis sativa* has been a subject of scientific interest for over 50 years (1). Moreover, it has been used for recreational and medical purposes for thousands of years. The plant comprises about 100 phytocannabinoids, which are C_21_ terpenophenolic constituents (2). Nowadays, the most-studied phytocannabinoids are: Δ^9^- tetrahydrocannabinol (Δ^9^-THC), Δ^9^-tetrahydrocannabivarin (Δ^9^-THCV), cannabinol (CBN), cannabidiol (CBD), cannabidivarin (CBDV), cannabigerol (CBG), and cannabichromene (CBC) (1). So far, many studies have shown therapeutic properties of the above mentioned *Cannabis* compounds. Therefore, the aim of the current review is to focus on the emerging potential of CBD and other phytocannabinoids, which act as novel therapeutic agents in obesity treatment.

## The Expanded Endocannabinoid System (ECs)

The canonical endocannabinoid system consists of the endocannabinoids (ECs), enzymes responsible for their production and metabolism as well as specific receptors (3). The most studied ECs, i.e., anandamide (AEA) and 2-arachidonoylglycerol (2-AG), are bioactive lipid mediators derived from long-chain polyunsaturated fatty acids (4, 5). It is known that they are released on demand and act through cannabinoid CB_1_ and CB_2_ receptors, which are G-protein-coupled receptors (GPCRs) (4–7). Interestingly, in the past the CB_1_ receptor was thought to be present only in the brain structures, whereas the CB_2_ receptor expression was limited to immune cells (8). However, recent research revealed that both CB_1_ and CB_2_ receptors are expressed in the brain and peripheral tissues, including the liver, skeletal muscle, heart, gut, bones, and adipose tissue (9–13). ECS mediators, AEA and 2-AG, are degraded by two enzymes: fatty acid amide hydrolase (FAAH) and monoacylglycerol lipase (MAGL), respectively (14). In addition the existence of many fatty acid-derived mediators, such as *N*-palmitoyl-, *N*-oleoyl-ethanolamine (PEA, OEA), 2-oleoyl-, 2-linoleoyl-glycerol (2-OG, 2-LG), and 2-arachidonoyl glyceryl ether (2-AGE, noladin ether), has been discovered recently (more than 100) (15, 16). These several congeners often share common molecular targets and are inactivated by the same enzymes as ECs. Among these novel targets we include some orphan GPCRs such as GPR55, thermosensitive transient receptor potential (TRP) channels and peroxisome proliferator-activated receptors α and γ (PPARα and PPARγ) (17). The aforementioned discoveries have extended the concept of this inner signaling system from ECS itself to the expanded ECS or endocannabinoidome (eCBome). The expanded ECS is involved in controlling various processes, including appetite, energy balance, metabolism, thermogenesis, inflammation, nociception as well as regulation of stress, and emotions (18). Recent data have shown that different phytocannabinoids interact with several components of endocannabinoidome by affecting either cannabinoid receptors (to a lesser extent), non-cannabinoid receptor targets and/or enzymes involved in the metabolism of endogenous ligands (11, 15). Hence, the interest on their regulatory mechanisms and a potent therapeutic action has risen greatly in the last few years.

### Δ^9^-Tetrahydrocannabinol (Δ^9^-THC)

**Figure 1:**
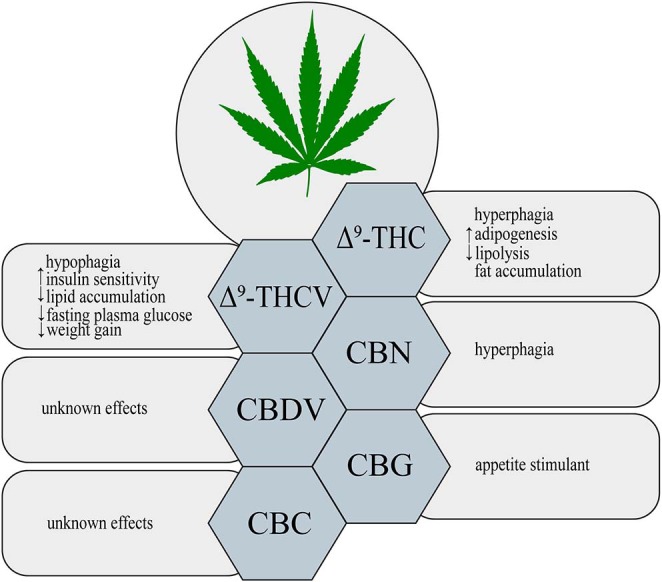
Metabolic effects of different phytocannabinoids. ↑, increase; ↓, decrease; Δ^9^-THC, Δ^9^- tetrahydrocannabinol; Δ^9^-THCV, Δ^9^-tetrahydrocannabivarin; CBN, cannabinol; CBDV, cannabidivarin; CBG, cannabigerol; CBC, cannabichromene.

Δ^9^-THC is a major psychoactive constituent of *Cannabis sativa* (1). It was shown that Δ^9^-THC acts as a partial agonist of both CB_1_ and CB_2_ receptors (19). Throughout such activation Δ^9^-THC is able to trigger many different physiological processes, i.e., regulation of gastrointestinal, liver and cardiovascular functions, pain perception along with modulation of neurotransmitters release in the nervous system (20, 21). Now, it is clearly established that, Δ^9^-THC exerts a well-known psychoactive effect, which is mediated by the activation of CB_1_ receptor in the central nervous system (CNS) (22). Additionally, Δ^9^-THC by activating CB_1_ receptors, located in the limbic (enhancing motivational properties of food) and hypothalamic (increasing appetite) structures, causes the orexigenic effects (Figure 1) (23). Apart from the above implications, Δ^9^-THC has also the ability to bind to eCBome receptors including GPR55 (G protein-coupled receptor 55), 5- HT_3A_ (serotonin receptor subunit), TRPV2, 3, 4 receptors (transient receptor potential channels of vanilloid type 2, 3, 4), which produce some of its pharmacological effects (22). Lauckner et al. revealed that Δ^9^-THC activates the GPR55 receptor in HEK293 and CHO cells, which resulted in increased intracellular calcium level (24). Another study conducted on HEK293 cells showed that Δ^9^-THC acts as a 5- HT_3A_ receptor antagonist, whereas several reports demonstrated that Δ^9^-THC has agonistic effects on the TRPV2, 3, 4 channels (25–28). Furthermore, Δ^9^-THC was reported to exhibit a beneficial impact on the regulation of insulin sensitivity in the insulin resistant adipocytes. The authors have demonstrated that natural extract containing Δ^9^-THC decreased the TAGs content and improved the glucose uptake in the insulin resistant 3T3-L1 cells in a concentration-dependent manner (29). In the same study it was shown that the level of tumor necrosis factor alpha (TNF-α) in the 3T3-L1 cells was substantially decreased in the presence of Δ^9^-THC, which also improved sensitivity of the cells to insulin (29). The above mentioned studies also revealed that in a differentiated 3T3-L1 cells Δ^9^-THC treatment enhanced glucose transporter type 4 (GLUT4) and insulin receptor substrate 1 and 2 (IRS-2) gene expressions, which play key roles in the insulin signaling pathway (29). However, we have to keep in mind that in the above experimental models natural extract were applied and no information upon their purity was provided, therefore, it can be considered as the limitation of such studies in the face of stating pharmacological properties of examined substances.

On the other hand, in comparison to the well-documented orexigenic effect of Δ^9^-THC, many studies have also shown a potential anti-obesity effect of this phytocannabinoid derivative. Cluny et al. (30) using the *in vivo* model have demonstrated that chronic administration of Δ^9^-THC to diet-induced obesity (DIO) mice for 3 and 4 weeks treatment (intraperitoneal injections of Δ^9^-THC in a dose of 2 or 4 mg/kg and vehicle) prevented weight gain due to reduced energy intake (30). This effect might have been associated with the fact that Δ^9^-THC is a partial agonist of CB_1_ and CB_2_ receptors. Since, Δ^9^-THC does not produce maximal stimulation of the above receptors, the background of such changes could result from blocking endogenous full agonist, i.e., anandamide from binding to CB_1_ receptor, especially, when the endocannabinoid tone is high (e.g., obesity) (30, 31). However, potential positive influence of Δ^9^-THC in the prevention and treatment of obesity requires further investigation.

### Δ^9^-Tetrahydrocannabivarin (Δ^9^-THCV)

Δ^9^-THCV is a propyl Δ^9^-THC analog, which binds to the cannabinoid CB_1_ and CB_2_ receptors (32). So far, data indicate that Δ^9^-THCV is a neutral antagonist at low doses and agonist at high doses of CB_1_ and CB_2_ receptors (2, 32, 33). Little evidence exists upon the activation of various eCBome receptors by Δ^9^-THCV. Nevertheless, it has been established that Δ^9^-THCV acts as an agonist of human TRPV1, rat TRP channels of ankyrin type-1 (TRPA1) and TRPV2, 3, 4 channels as well as an antagonist of rat's TRP channel of melastatin type-8 (TRPM8) (26). In one study it was demonstrated that Δ^9^-THCV activated 5-HT_1A_ receptor in the rat brain and human 5-HT_1A_ receptor-transfected CHO (Chinese hamster ovary) cells, and thereby exerted antipsychotic effects (34). Interestingly, recent research has shown that this non-psychotropic phytocannabinoid has a potential therapeutic effect in the treatment of diabetes associated with obesity. Wargent et al. (35) revealed that Δ^9^-THCV increased insulin sensitivity and improved glucose tolerance in DIO mice as well as genetically obese *ob/ob* mice (Figure 1). However, it did not substantially affect food intake and body weight gain in the above models, whereas body fat content was decreased (35). In the same experiment, the authors investigated the impact of Δ^9^-THCV on the insulin resistant C_2_C_12_ myotubes and HL-5 hepatocytes showing that Δ^9^-THCV restored intracellular insulin signaling pathway (35). Other studies have also displayed that Δ^9^-THCV (1–10 μM) decreased lipid accumulation in the *in vitro* model of hepatosteatosis in HHL-5 (Human Hepatocyte Line 5) cells and adipocytes (3T3-L1 cells) (36). According to the above results, a pilot study in patients with type 2 diabetes showed that Δ^9^-THCV reduced fasting plasma glucose with parallel improvement in β-cell function as well as increased Apo A (apolipoprotein A) and adiponectin concentrations compared with placebo, which was well-tolerated in patients (37). Nonetheless, further research is necessary to confirm the potential therapeutic properties of Δ^9^-THCV in the treatment of obesity, metabolic syndrome and type 2 diabetes.

### Cannabinol (CBN)

An oxidized metabolite of Δ^9^-THC, cannabinol, was isolated in 1896 by Wood and colleagues (38). CBN is a non-enzymatic oxidative breakdown product of Δ^9^-THC due to aging or light exposure (32). It was shown that CBN binds to cannabinoid receptors CB_1_ and CB_2_ in the peripheral organs and central nervous system (CNS), and therefore, it exerts a weak psychoactive activity (39). CBN also displays the ability to bind to expanded ECS receptors including agonistic and antagonistic effects on TRPA1 and TRPM8 channels, respectively (26). So far, studies focusing on the impact of CBN on obesity and feeding behavior have been limited. Only in one of the recent studies it has been demonstrated that CBN increased food intake in rats in a dose-dependent manner (Figure 1) (40). Probably, the hyperphagic CBN's effect was induced by its interaction with CB_1_ receptor in CNS (40) but it should be confirmed by additional experiments including for example gene silencing or knockout animals. Nevertheless, further experiments are required to completely characterize the role of CBN in food consumption and body weight control.

### Cannabidivarin (CBDV)

Among phytocannabinoids we can find a propyl analog of CBD, cannabidivarin, which exerts non-psychotropic properties (32). CBDV has a weak affinity for cannabinoid receptors CB_1_ and CB_2_ with simultaneous stronger activation of other molecular targets related with the ECS (41). Predominantly, anticonvulsant and other therapeutic CBDV's properties are associated with activation of TRP channels (TRPV1, 2 and TRPA1) and TRPM8 inhibition (26, 42). Another pharmacological target for CBDV is GPR55 receptor, where observed effect is inhibitory (43). However, currently there is insufficient research on the ground of CBDV's effects on obesity, insulin resistance and other metabolic disturbances (Figure 1).

### Cannabigerol (CBG)

Another phytocannabinoid, which lacks psychotropic properties and occurs only in trace amounts in *Cannabis*, is cannabigerol (41). *Cannabis* plants produce cannabigerolic acid (CBGA), which is a direct precursor of cannabinoids: Δ^9^-THC, CBC and CBD (44, 45). Preliminary studies, using plasma membranes isolated from the CHO and HEK-293 cells expressing human CB_1_ and CB_2_ receptors, have shown that CBG acts as a partial agonist of both cannabinoid receptors (46). Similarly to other phytocannabinoids CBG has an ability to interact with endocannabinoidome receptors, such as TRPV1, 2, TRPA1, and TRPM8 channels (26). Moreover, one study has provided for the first time evidence upon activation of α_2_-adrenoreceptors and 5-HT_1A_ receptor blockage by CBG in the vas deferens and brain, respectively (47). Furthermore, recent investigation has revealed that CBG serves as a novel appetite stimulant in rats and importantly, no side effects were observed during its administration (Figure 1) (48). However, extensive research is necessary to determine a more detailed mechanisms of CBG action with respect to metabolic diseases.

### Cannabichromene (CBC)

The discovery of cannabichromene was reported by Izzo et al. (32). It is a minor component of *Cannabis* plant, which presents low affinity for CB_1_ and CB_2_ receptors (49, 50). Nonetheless, researchers discovered another molecular target of CBC, i.e., TRP channels. De Petrocellis et al. revealed that CBC is the most potent agonist of the TRPA1 channels (26). However, it has also the ability to activate in a lower potency, TRPV3 and TRPV4 channels, and additionally inhibits TRPM8 receptors, but to a much lesser extent (26). Although CBC exhibits different medical properties, its influence on metabolism and obesity has not been found, yet (Figure 1).

### Cannabidiol (CBD)

**Figure 2:**
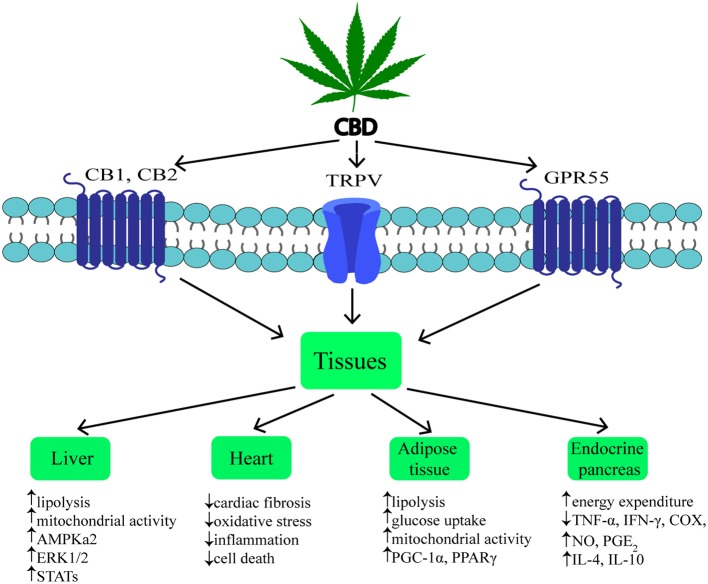
Mechanism and effects of CBD action on various tissues during obesity. ↑, increase; ↓, decrease; CBD, cannabidiol; CB_1_, cannabinoid receptor 1; CB_2_, cannabinoid receptor 2; TRPV1, transient receptor potential channel of vanilloid type 1; GPR55, G protein coupled receptor 55; AMPKa2, catalytic subunit of AMP-activated protein kinase; ERK1/2, extracellular signal-regulated kinase; STATs, signal transducers and activators of transcription; PGC-1α, peroxisome proliferator-activated receptor gamma coactivator 1-alpha; PPARγ, peroxisome proliferator-activated receptor gamma; TNF-α, tumor necrosis factor alpha; IFN-γ, interferon gamma; COX, cyclooxygenase; NO, nitrogen oxide; PGE_2_, prostaglandin E2; IL-4, interleukin 4; IL-10, interleukin 10.

Cannabidiol (CBD) is one of the most common non-psychotropic constituent of *Cannabis* plant (this depends on the cannabis strain). It was revealed that CBD induces a wide-range of pharmacological effects through different mechanisms. CBD functions as a negative allosteric modulator of CB_1_ receptor (51). Therefore, it has therapeutic potential in the treatment of the central nervous system diseases (neurodegenerative diseases, epilepsy, anxiety, and depression) without concurrent psychotic side effects (2, 52, 53). Furthermore, it was indicated that CBD is able to block CB_1_ receptor, thereby producing anti-obesity effects. Otherwise, CBD unexpectedly exhibited a high affinity for CB_2_ receptor as an agonist or inverse agonist depending on the research model (*in vitro* or *in vivo*) (54, 55). An interesting result of the latest studies was the fact that CBD has greater affinity for various eCBome receptors, including GPR55, α1-adrenoreceptors, 5-HT_1A_, TRPV channels and PPARγ (Figure 2) (19, 26, 42, 56, 57). Hegde et al. as well as Esposito et al. reported that CBD significantly induced the transcriptional activity of PPARγ (58, 59). It is known that PPARγ is an interesting therapeutic target due to its crucial role in regulating glucose homeostasis, lipoprotein metabolism, and inflammation (60, 61).

Moreover, recent research has focused on the therapeutic properties of CBD, including anti-inflammatory, anti-oxidant, anti-tumor, anti-convulsant and neuroprotective effects (62, 63). In the light of the above mentioned properties, CBD emerges as a potential therapeutic agent, which can be used in the treatment of diabetes and its complications, obesity, ischemia, neurodegenerative diseases as well as pain relieving and depression. Although CBD's anti-inflammatory and neuroprotective properties are well-confirmed, there are few studies that have investigated anti-obesity effects of this compound. Wierucka-Rybak et al. have examined the effect of CBD on food intake, food preferences and weight gain in rats (64). The authors administered CBD (3 mg/kg) for 3 days to rats maintained on a standard diet (SD), high fat diet (HFD), or free choice diet (FC; high sucrose). The authors revealed that CBD injections in the case of rats being on the HFD resulted in an increase in body weight despite significantly reduced food intake. On the other hand, in rats fed FC diet CBD did not cause significant change in food consumption and body weight (64). Previous studies investigating the impact of CBD on food intake showed contradictory results. One study demonstrated CBD-induced (2.5 and 5 mg/kg) decrease in body weight gain in rats (54), while other studies have shown no significant impact on food intake and body weight in mice and rats (65, 66). Importantly, pilot studies were conducted to investigate the effect of CBD on glycemic and lipid parameters in patients with type 2 diabetes (37). The findings from these studies demonstrated that CBD did not produce any improvement in glycemic and lipid control despite producing eligible changes in gut hormones (GIP—glucose dependent insulinotropic peptide) and adipokines (resistin) concentrations (37). Probably, the reason for the lack of therapeutic effects seen during CBD administration could have been too low dosage used in the study. The anti-epileptic properties of CBD were investigated in many preclinical studies and in randomized trials (67–71). Data obtained from these studies confirmed CBD's anti-seizure properties along with a good tolerance profile. Currently, the first cannabis based medicine containing CBD/Δ^9^-THC combination for the multiple sclerosis related spasticity treatment (Sativex/Nabiximols; GW Pharmaceuticals) has been approved in numerous countries, including Australia, Canada, New Zealand and in most European Union countries (72). Furthermore, a drug composed solely of CBD (Epidiolex; GW Pharmaceuticals) has been approved in June 2018 in the United States for the treatment of Dravet and Lennox-Gastaut syndromes in children (52, 73, 74).

## Overactivation of the ECS in Obesity

Obesity and coexisting disorders such as insulin resistance, hypertension, and hypertriglyceridemia lead to the development of metabolic syndrome and type 2 diabetes (75). Whenever, the excessive fatty acids (FAs) consumption takes place, simultaneously we can observe an increased differentiation of pre-adipocytes to mature adipocytes with subsequent stimulation of their growth as well (76, 77). Over time, at a later stage of obesity development, adipocytes are overloaded, which results in the accumulation of lipids in other tissues such as liver, skeletal, and cardiac muscles (60, 78). Paralell excessive fat accumulation can be observed in the liver or cardiac muscle, which contributes to the development of liver steatosis and cardiomyopathy, respectively (78). Many scientists are looking for new therapeutic strategies, including expanded ECS, which can be a useful tool in preventing and treating the above mentioned diseases. Therefore, the components of eCBome are emerging as potent therapeutic targets due to their well-established role in the regulation of food consumption and energy balance as well as lipid and glucose metabolism (79, 80). Literature data indicated, that ECS is upregulated during obesity and associated diseases (81–84). It is well-confirmed that the level of endogenous cannabinoids in the above mentioned conditions is increased, i.e., in CNS, adipose tissue, pancreas, skeletal muscle, kidney, liver, and blood of obese rodents and humans (76, 84–87). The cause of such ECS overactivation may be due to enhanced synthesis of ECs or their reduced degradation as well as overexpression of the cannabinoid receptors (88, 89). Various studies have shown upregulated levels of 2-AG in both different organs and serum during obesity and hyperglycemia, which was correlated with body fat content, visceral fat mass and fasting plasma triacylglyceride and insulin concentrations (84, 86, 87, 90). On the other hand, the reverse situation was described for ECs in the liver of DIO mice, where the hepatic levels of AEA were increased in animals fed HFD, while no significant difference in 2-AG liver levels was observed (91). Accordingly, Kimberly et al. revealed substantial association between AEA level and body mass index (BMI) value, which was an argument for making it a biomarker of NASH (non-alcoholic steatohepatitis) (92). In contrast with the above results, other studies have shown that patients with NAFLD (non-alcoholic fatty liver disease) had significantly increased levels of 2-AG without any change in AEA levels (93). Hence, it has been proposed to attenuate overactivation of ECS as a new approach for the treatment of obesity and its coexisting disorders. Such mechanism was used by researchers to create an anti-obesity drug (rimonabant; SR141716A), which was the first selective antagonist of CB_1_ receptors expressed in the brain and different peripheral organs/tissues controlling energetic homeostasis of the body (liver, muscle, adipose tissue, etc.) (94–96). Several studies have confirmed the beneficial effect of rimonabant on cardiometabolic risk markers, body weight as well as lipid and glucose parameters (97–99). However, rimonabant (Acomplia® Sanofi-Aventis) treatment turned out to be harmful so that it was forbidden in 2009 due to its adverse psychotropic side effects (100). This contributed to the invention of peripherally restricted CB_1_ receptor antagonists with limited brain penetration. Many studies investigating the effects of these antagonists (inverse agonists), such as AM6545, JD5037, have shown their positive effects against obesity in preclinical studies (101–103). For instance, AM6545 (10 mg/kg per day) treatment in DIO and genetically obese (*ob/ob*) mice attenuated obesity-related glucose intolerance, insulin resistance, dyslipidemia and reversed hepatic steatosis (104). Accordingly, Tam et al. demonstrated the hypophagic and weight-reducing effects of JD5037 (3 mg/kg per day, 7 days) in DIO mice but not in *ob/ob* and *db/db* mice, indicating leptin-dependent action (102). Importantly, JD5037 treatment resulted in the attenuation of hyperglycemia, hyperinsulinemia, insulin resistance, and reduction of hepatic triacylglycerols in all the above mentioned strains (102). We can conclude that peripherally restricted CB_1_ receptor antagonists have great therapeutic potential in the treatment of obesity.

## Specific Sites of CBD Actions Related to Obesity

### Liver

In view of the increasing prevalence of obesity, type 2 diabetes and metabolic syndrome, the development of liver diseases occurs more often. The above mentioned diseases are associated with excessive fat accumulation resulting from upregulated FA influx and *de novo* lipid synthesis in different cells, e.g., hepatocytes (105). Alterations in the hepatic fatty acid oxidation are highly related with the aforementioned metabolic disturbances, which were shown in many experimental models together with discrepancies in this field. Studies conducted on NASH and liver steatosis patients showed an increase (106, 107), decrease (108), or no change (109) in the fatty acid oxidation status. On the other hand, the expected consequence of lipid accumulation in the liver would be simultaneous enhancement in hepatic oxidation of fatty acids, thus, it seems that certain factors can also influence this process as well.

In previous studies it was shown that, hepatocytes produce endocannabinoids, AEA and 2-AG, and possess CB_1_ and CB_2_ receptors (80, 91). A study carried out by Liu et al. revealed that liver specific CB_1_ receptor knockout mice were protected from the development of both insulin resistance and hepatosteatosis but not obesity (55). Silvestri et al. revealed positive influence of CBD on the liver, namely the reduction of intracellular lipid content in the *in vitro* hepatosteatosis model, possibly by enhancing lipolysis and mitochondrial activity through increased fatty acids oxidation (36). The authors have shown that CBD increases the expression of selected proteins involved in upregulating lipid metabolism, i.e., catalytic subunit of 5′AMP-activated protein kinase (AMPKa2), extracellular signal-regulated kinase (ERK1/2) along with signal transducers and transcription activators (STATs) in hepatocytes (Figure 2, Table 1) (36). Concomitantly, the authors observed that CBD enhanced the level of glutathione (GSH), adenosine triphosphate (ATP), and nicotinamide adenine dinucleotide (NAD), which supported the assumption that CBD increases intracellular lipolysis and mitochondrial activity (36). Additionally, the study carried out by Wang's research group (119) indicated, in a murine model, an impact of CBD on alcohol-induced liver injury. The obtained results displayed that CBD attenuates liver steatosis (decreased triacylglyceride and fat droplet accumulation), inflammatory response [reduced the alcohol-feeding induced mRNA expressions of interleukin 1beta (IL1β), TNF-α and monocyte chemoattractant protein 1 (MCP1)], oxidative/nitrative stress (reduced lipid peroxidation, expression of reactive oxygen species generating enzyme—NADPH oxidase 2 (NOX2) and 3-nitrotyrosine production) and neutrophil infiltration in the liver (Table 1). This also confirmed the well-known, whole body anti-inflammatory and antioxidant properties of CBD (119).

**Table 1:** Summary of the effects of CBD on different tissues during obesity.

**Organ**	**ECS overactivation**	**CBD treatment**	**References**
Brain	↑food intake, ↑motivation for palatable food	Unknown	(9, 23)
Liver	↑lipogenesis, ↑fibrogenesis, ↑hepatic apoptosis, ↑hepatocyte proliferation, ↓AMPK, ↑dyslipidemia, ↑steatosis, ↑fibrosis, ↑insulin resistance	↓liver enzymes level, ↓steatosis, ↓inflammation, ↓liver damage, ↓pro-inflammatory cytokines	(83, 110–112)
Gastrointestinal tract	↑food assimilation, ↑permeability, ↓satiety, ↓motility, ↓inflammation	↑GIP level, ↓resistin concentration	(37, 110, 111)
Adipose tissue	↑lipogenesis, ↑glucose uptake, ↓FA oxidation, ↓lipolysis, ↓mitochondrial biogenesis, ↓UCP1, ↑storing capacity, ↑insulin resistance, ↓thermogenesis	↑lipolysis, ↑thermogenesis, ↑PGC-1α, UCP1, PPARγ expression, ↓lipogenesis	(31, 113)
Endocrine pancreas	↑/↓insulin secretion, ↑insulin resistance, ↑Bad protein activation, ↑β-cell death	↑anti-inflammatory cytokines ↓pro-inflammatory cytokines	(114–116)
Muscles	↓insulin signaling ↓glucose uptake, ↓AMPK	Unknown	(9, 83)
Heart	↑AT1 receptor expression, ↑ROS generation, ↑MAPK activation, ↑AGEs accumulation ↑hypotension, bradycardia, negative inotropy, ↑inflammation, ↑apoptosis, ↓inflammatory response	↓infarct size, ↓infiltrating leukocytes, ↓platelet aggregation, ↓inflammation, ↓fibrosis	(11, 117, 118)

*↑, increase; ↓, decrease; CBD, cannabidiol; AMPK, AMP-activated protein kinase; GIP, glucose dependent insulinotropic peptide; UCP1, uncoupling protein 1; PGC-1α, peroxisome proliferator-activated receptor gamma coactivator 1-alpha; PPARγ, peroxisome proliferator-activated receptor gamma; AT1, angiotensin II receptor type 1; ROS, reactive oxygen species; MAPK, mitogen-activated protein kinase; AGEs, advanced glycation end products*.

### Adipose Tissue

Obesity is associated with chronic low-grade inflammatory state and excessive fat accumulation (120). The expression of CB_1_ and CB_2_ receptors, other eCBome molecular targets (i.e., TRPV1,GPR55) and ECs enzymes level in the visceral and subcutaneous adipose tissue has been found (121, 122). These additional receptors are the most promising target for major non-psychogenic constituent of *Cannabis plant*, which has well-established anti-inflammatory effects and potential anti-obesity properties (123). Silvestri et al. (36) pointed out that CBD, depending on the time and dose, reduced triacylglycerols accumulation in 3T3-L1 adipocytes treated with oleic acid (OA). These results emphasize a potential role of CBD on lipolysis induction (Table 1). However, more research is necessary to investigate the exact mechanism of this phenomenon (36). Similarly, Ramlugon et al. (124) revealed that CBD treatment, in a time-dependent manner, induced the mitochondrial activation and increased oxygen consumption, which may be an explanation for the reduced fat accumulation in adipocytes despite of increased glucose uptake (Figure 2) (124). Additionally, recent research has shown that CBD inhibited weight gain in rats subjected to high fat diet (HFD) for 14 days and this effect was probably mediated by CB_2_ receptor (54). The authors confirmed the above effect by using a selective CB_2_ antagonist-AM630, which prevented the reduction in weight gain due to CBD treatment (54). However, additional studies are required to uncover the mechanisms by which CBD induces the above metabolic changes in adipocytes.

### Endocrine Pancreas

The pancreas plays a pivotal role in the blood glucose homeostasis and whole body metabolism through insulin and glucagon secretion (125). In obesity, secretion of the above hormones is dysregulated. To date, the expression of cannabinoid receptors within the endocrine pancreas has been shown (114, 121). However, inconsistent data have been obtained regarding the expression of CB_1_ and CB_2_ receptors in the pancreas islet cells. Starowicz et al. (121) demonstrated the expression of CB_1_ receptor in glucagon-secreting α-cells of mice, while CB_2_ receptor was found inside mouse α-cells and insulin-secreting β-cells. Similarly, in the study conducted on human samples it was determined that CB_1_ receptor was present in glucagon-secreting α-cells and in a lesser extent in insulin-secreting β-cells, whereas CB_2_ was only located in somatostatin-secreting delta cells (114). On the other hand, other studies indicated that β-cells exhibited the expression of both cannabinoid receptors or only CB_2_ receptor (84, 115). Interestingly, Levendal et al. (126) investigated the effect of CBD on β-cell function in obese rats. The authors found that in a rat model of diet-induced obesity (DIO), *cannabis* extract treatment reduced the weight gain (mainly fat depots) and increased energy expenditure through upregulation of protein kinase B (PKB), mitochondrial uncoupling protein 2 (UCP2), and glucose transporter 2 (GLUT2) expression in pancreatic β-cells (rats were injected subcutaneously every second day for 28 days; the first five treatments containing an equivalence of 5 mg THC/kg body weight and the remaining treatments an equivalence of 2,5 mg THC/kg body weight) (126). Moreover, studies conducted by Weiss et al. have shown that CBD treatment (mice were administered 10–20 intraperitoneal injections at a dose of 5 mg/kg CBD) decreased the frequency of type 1 diabetes incidence and development in a model of non-obese diabetic (NOD) mice (116). Furthermore, the authors reported that CBD reduced plasma level of the pro-inflammatory cytokines, i.e., TNF-α and interferon gamma (IFN-γ) together with inflammation mediators, such as nitric oxide (NO), cyclooxygenase (COX), and prostaglandin E2 (PGE2). Whereas, anti-inflammatory cytokines—interleukins IL-4, IL-10 levels were increased (Figure 2, Table 1). Hence, reduced pancreas islets inflammation was observed in the histological examination as well as lower β-cell destruction (116). Concluding, it is likely that CBD has the ability to prevent and reduce the pancreatic damage associated with obesity and insulin resistance.

### Cardiac Muscle

Chronic inflammatory state associated with obesity reduces the ability of the adipose tissue to store incoming plasma-borne fatty acids and leads to their accumulation in other organs, including cardiac muscle (127). Then, the local inflammatory state, oxidative stress and hyperinsulinemia can develop and progress induction of insulin resistance in the heart muscle (128). Currently, many studies are focused on the effect of CBD on the lipid and glucose metabolism in the heart, which is dysregulated during obesity and subsequently can lead to the cardiomyopathy and cardiac contractile dysfunction development (117). The impact of CBD on myocardial dysfunction, oxidative/nitrative stress, inflammation, and cell death has been recently investigated by Rajesh and coworkers (117). They used a primary human cardiomyocytes subjected to a high glucose concentration and a mouse model of type 1 diabetic cardiomyopathy. It was demonstrated that CBD reduced cardiac fibrosis, myocardial oxidative/ nitrative stress, inflammation and cell death in diabetic hearts (in the first set of experiments, 1 week diabetic mice were treated with CBD 1, 10 or 20 mg/kg intraperitoneal injections for 11 weeks; in the second set of experiments 8 weeks diabetic mice were treated with CBD for 4 weeks) (**Figrue 2**, Table 1) (117). These effects were mediated by decreased intracellular adhesion molecule 1 (ICAM-1), vascular cell adhesion molecule 1 (VCAM-1), and TNF-α expression as well as reduced mitogen-activated protein kinase (MAPK) and nuclear factor-κB (NFκB) activation. Moreover, CBD attenuated the high glucose-induced enhanced ROS (reactive oxygen species) generation and cell death in human cardiomyocytes (117). These findings highlight the role of CBD in the prevention or treatment of diabetic complications.

## Conclusions

Overweight, insulin resistance and obesity emerged as leading health concerns all over the world. The above mentioned disturbances are characterized by excessive or abnormal fat accumulation, and are major risk factors for a number of chronic diseases, such as cardiovascular diseases, diabetes, and cancer. Currently, the non-psychotropic component of *Cannabis sativa*—CBD is in the center of interest, due to its well-established anti-inflammatory, anti-oxidant, anti-convulsant, anti-psychotic and potential anti-obesity properties. Many studies indicated that CBD affects both lipid and glucose metabolism through the action on various receptors as well as several metabolites. From the existing data, we can conclude that CBD has the promising potential as a therapeutic agent and might be effective in alleviating the symptoms of insulin resistance, type 2 diabetes and metabolic syndrome.

## Author Contributions

PB participated in the design of the work, drafted the manuscript, prepared figures and tables, and approved final version submitted. EH-S helped to draft the manuscript and approved final version submitted. AC participated in the design of the study, revised manuscript, and approved final version submitted. All authors agreed to be accountable for all aspects of the work.

### Conflict of Interest

The authors declare that the research was conducted in the absence of any commercial or financial relationships that could be construed as a potential conflict of interest.
